# Open Repair of Primary Versus Recurrent Male Unilateral Inguinal Hernias: Perioperative Complications and 1-Year Follow-up

**DOI:** 10.1007/s00268-015-3325-9

**Published:** 2015-11-18

**Authors:** F. Köckerling, A. Koch, R. Lorenz, W. Reinpold, M. Hukauf, C. Schug-Pass

**Affiliations:** Department of Surgery and Center for Minimally Invasive Surgery, Academic Teaching Hospital of Charité Medical School, Vivantes Hospital, Neue Bergstrasse 6, 13585 Berlin, Germany; Hernia Center Cottbus, Gerhard-Hauptmann-Strasse 15, 03044 Cottbus, Germany; 3Surgeons Practice, Klosterstrasse 34/35, 13581 Berlin, Germany; Department of Surgery and Hernia Center, Wilhelmsburg Hospital Gross-Sand, Gross-Sand 3, 21107 Hamburg, Germany; StatConsult GmbH, Halberstädter Strasse 40 a, 39112 Magdeburg, Germany

## Abstract

**Introduction:**

The recommendation in the European Hernia Society Guidelines for the treatment of recurrent inguinal hernias is to modify the technique in relation to the previous technique, and use a new plane of dissection for mesh implantation. However, the registry data show that even following previous open suture and mesh repair to treat a primary inguinal hernia, open suture and mesh repair can be used once again for a recurrent hernia. It is therefore important to know what the outcome of open repair of recurrent inguinal hernias is compared with open repair of primary inguinal hernias, while taking the previous operation into account.

**Patients and methods:**

In the Herniamed Registry, a total of 17,594 patients with an open primary or recurrent unilateral inguinal hernia repair in men with a 1-year follow-up were prospectively documented between September 1, 2009 and August 31, 2013. Of these patients, 15,274 (86.8 %) had an open primary and 2320 (13.2 %) open recurrent repair. In the unadjusted and multivariable analyses, the dependent variables were intra- and postoperative complications, reoperations, recurrences, pain at rest, pain on exertion, and pain requiring treatment.

**Results:**

Open recurrent repair compared with the open primary operation is a significant influence factor for higher intraoperative (*p* = 0.01) and postoperative (*p* = 0.05) complication rates, recurrence rate (*p* < 0.001), and pain rates (*p* < 0.001). With regard to repair of recurrent inguinal hernia, previous open mesh repair was associated with the least favorable outcome, and with the highest odds ratio, for all outcome criteria. Open recurrent repair following previous endoscopic operation presented the least risk for postoperative complications, complication-related reoperations, and re-recurrences. The pain rates identified on follow-up after open recurrent repair were lower following previous open suture operation compared with following open and endoscopic mesh repair.

**Summary:**

A significantly less favorable perioperative and 1-year follow-up outcome must be expected for open repair of recurrent inguinal hernia in comparison with open primary inguinal hernia repair. After open recurrent repair, the most favorable perioperative complication and recurrence rates were identified following previous endoscopic repair, and the lowest pain rates following previous open suture repair. Open recurrent repair following previous open mesh operation was associated with the highest risks for perioperative complications, re-recurrences, and pain.

## Introduction

The recommendation in the European Hernia Society Guidelines for the treatment of recurrent hernias is to modify the technique in relation to the previous technique, and use a new plane of dissection for mesh implantation [1, 2]. However, the registry data show that even following previous open suture and mesh repair to treat the primary inguinal hernia, open suture and mesh repair are used once again for a recurrent hernia [2], despite meta-analyses and systematic reviews having identified advantages for endoscopic repair [3–7]. For example, based on data from the Swedish Hernia Registry following previous inguinal hernia repair in Lichtenstein or plug technique, the recurrence was repaired in 32.9 % of cases once again in Lichtenstein technique, in 26.4 % in endoscopic technique, in 16.5 % as plug and patch procedure, in 13.8 % in open preperitoneal technique, and in 2.7 % of cases in suture technique [2]. That was no doubt due to the fact that the skill needed for endoscopic repair of recurrent inguinal hernias was not always assured. Where surgeons had used an open technique to repair 95 % of primary inguinal hernias, then more than 90 % of recurrences were also repaired using an open procedure [8]. That was also true when using mesh repair for the primary inguinal hernia operation [9]. Comparison of 75 recurrences with 287 primary inguinal hernias repaired in Lichtenstein technique identified a tendency toward better outcomes for primary inguinal hernia patients [10]. Accordingly, it is unlikely that in the future either the majority of recurrent inguinal hernias will be repaired in endoscopic technique following previous open suture or mesh repair. This means that it is all the more important to know the outcome of open repair of recurrent inguinal hernias compared with open repair of primary unilateral inguinal hernias in order to make patients aware of the corresponding risk during the informed consent discussion.

The heterogeneous nature of recurrent hernias makes controlled trials in this field difficult and time-consuming, particularly when the previous repair has to be taken into consideration [2]. Large national hernia registers are a valuable way of obtaining information on recurrent groin hernia surgery.

Based on data from the Herniamed Registry, this present paper now compares open repair of recurrent inguinal hernias with open repair of primary inguinal hernias. Only male unilateral hernias are taken into account. The target criteria used are the perioperative complications as well as recurrence and pain rates on 1-year follow-up.

## Patients and methods

The Herniamed Registry is a multicenter, internet-based hernia registry [11] into which 425 participating hospitals and surgeons engaged in private practice (Herniamed Study Group) had entered data prospectively on their patients who had undergone hernia surgery. All postoperative complications occurring up to 30 days after surgery are recorded. On 1-year follow-up, postoperative complications are once again reviewed when the general practitioner and patient complete a questionnaire. This present analysis compares the prospective data collected for all male patients with a minimum age of 16 years, who had undergone elective primary or recurrent unilateral inguinal hernia repair using open mesh (Lichtenstein, Plug, Gilbert and TIPP) repair [12, 13].

In total, 17,594 patients were enrolled between September 1, 2009 and August 31, 2013. Of these patients, 15,274 (86.8 %) had open primary repair and 2320 (13.2 %) had open recurrent repair. All the patients had to have a 1-year follow-up (follow-up rate: 100 %).

The demographic- and surgery-related parameters included age (years), BMI (kg/m^2^), ASA score (I, II, III–IV), procedure (Lichtenstein, Plug, Gilbert and TIPP) as well as EHS classification (hernia type: medial, lateral, femoral, scrotal. Defect size: Grade I = < 1.5 cm, Grade II = 1.5–3 cm, Grade III > 3 cm) [14] and risk factors (nicotine, COPD, diabetes, cortisone, immunosuppression, etc.). Risk factors were dichotomized, i.e., “yes” if at least one risk factor is positive and “no” otherwise.

The dependent variables were intra- and postoperative complications rates, number of reoperations due to complications, as well as the 1-year results (recurrence rate, pain at rest, pain on exertion, and pain requiring treatment).

All analyses were performed with the Software SAS 9.2 (SAS institute Inc. Cary, NY, USA) and intentionally calculated to a full significance level of 5 %, i.e., they were not corrected in respect of multiple tests, and each *p* value ≤ 0.05 represents a significant result. To discern differences between the groups in unadjusted analyses, *χ*^2^ test was used for categorical outcome variables, and the ANOVA (analysis of variance) for continuous variables.

To rule out any confounding of data caused by different patient characteristics, the results of unadjusted analyses were verified via multivariable analyses in which, in addition to primary or recurrent operation, other influence parameters were simultaneously reviewed.

To identify influence factors in multivariable analyses, the binary logistic regression model for dichotomous outcome variables was used. Estimates for odds ratio (OR) and the corresponding 95 % confidence interval based on the Wald test were given. For influence variables with more than two categories, one of the latter forms was used in each as reference category. For age (years), the 10-year OR estimate and for BMI (kg/m^2^) the 5-point OR estimate were given. Results are presented in tabular form, sorted by descending impact.

## Results

### Unadjusted analysis

Open recurrent repair was performed for 1011 (43.6 %) patients following previous open suture repair, for 897 (38.7 %) patients following endoscopic mesh repair, and for 412 (17.7 %) patients after open mesh repair of the primary inguinal hernia (Table 1). The open procedures used for recurrent repair are shown in Table 2, together with the previous operation. Just as for the primary procedures listed here on the basis of the registry data, so too for recurrent repair was the Lichtenstein technique used most often, followed by the Plug, TIPP, and Gilbert techniques.

**Table 1:** Open primary and recurrent unilateral inguinal hernia repair in men with distribution of previous operations

	Primary		Recurrence *n* = 2.320 (13.2 %)						Total	
			Previous suture		Previous open mesh		Previous endoscopic mesh			
	*n*	%	*n*	%	*n*	%	*n*	%	*n*	%
Open	15274	86.8	1011	43.6	412	17.7	897	38.7	17594	100.0

**Table 2:** Procedures in open primary and recurrent unilateral inguinal hernia repair in men with distribution of previous operations

Procedure	Open primary		Previous suture		Previous open mesh		Previous endoscopic mesh		*p*
	*n*	%	*n*	%	*n*	%	*n*	%	
Lichtenstein	11545	75.6	732	72.4	232	56.3	818	91.2	<.001
Plug	1970	12.9	174	17.2	102	24.8	46	5.1	
TIPP	940	6.2	41	4.1	67	16.2	13	1.5	
Gilbert	819	5.3	64	6.3	11	2.7	20	2.2	

The following surgical procedures were used for open primary repair of unilateral inguinal hernias in men: Lichtenstein *n* = 11,545 (75.6 %), Plug *n* = 1970 (12.9 %), TIPP *n* = 940 (6.2 %), and Gilbert *n* = 819 (5.4 %) (Table 2).

Significant differences were seen with regard to mean age and BMI between open repair of primary and recurrent inguinal hernias (Table 3). That was also true for the patient-related (Table 4) and risk factors

**Table 3:** Age and BMI of patients with open primary and recurrent unilateral inguinal hernia repair in men

		Primary	Previous operation			*p*
			Suture	Open mesh	Endoscopic mesh	
Age (years)	mean ± STD	61.2 ± 15.7	63.3 ± 16.3	60.6 ± 15.9	59.3 ± 13.5	<0.001
BMI (kg/m²)	mean ± STD	25.9 ± 3.4	25.8 ± 3.3	25.9 ± 3.6	26.3 ± 3.6	0.001

**Table 4:** Patient-related factors in open primary and recurrent unilateral inguinal hernia repair in men

	Open primary		Previous suture		Previous open mesh		Previous endoscopic mesh		*p*
	*n*	%	*n*	%	*n*	%	*n*	%	
ASA score									<0.001
I	4942	32.4	244	24.1	124	30.1	257	28.7	
II	7110	46.5	499	49.4	209	50.7	502	56.0	
III/IV	3222	21.1	268	26.5	79	19.2	138	15.4	
Defect size									<0.001
I (<1.5 cm)	1603	10.5	159	15.7	81	19.7	151	16.8	
II (1.5–3 cm)	7742	50.7	507	50.2	204	49.5	493	55.0	
III (>3 cm)	5929	38.8	345	34.1	127	30.8	253	28.2	
EHS classification medial									<0.001
Yes	7819	51.2	546	54.0	249	60.4	518	57.8	
No	7455	48.1	465	46.0	163	39.6	379	42.3	
EHS classification lateral									<0.001
Yes	10068	65.2	579	57.3	221	53.6	452	50.4	
No	5206	34.1	432	42.7	191	46.4	445	49.6	
EHS classification femoral									<0.001
Yes	171	1.1	14	1.4	18	4.4	15	1.7	
No	15103	98.8	997	98.6	394	95.6	882	98.3	
EHS classification scrotal									<0.001
Yes	1012	6.6	52	5.1	11	2.7	12	1.3	
No	14262	93.3	959	94.9	401	97.3	885	98.5	

(Table 5). Highly significant differences (in each case *p* < 0.001) were noted for the categorical influence variables, i.e., ASA score, hernia size, EHS classification, and risk factors (Table 4, 5).

**Table 5:** Risk factors in open primary and recurrent unilateral inguinal hernia repair in men

Risk factor	Open primary		Previous suture		Previous open mesh		Previous endoscopic mesh		*P*
	*n*	%	*n*	%	*n*	%	*n*	%	
Total									
Yes	5631	36.9	418	41.4	141	34.2	275	30.7	<.001
No	9643	63.1	593	58.7	271	65.8	622	69.3	
COPD									
Yes	1202	7.9	113	11.2	36	8.7	66	7.4	0.002
No	14072	92.1	898	88.8	376	91.3	831	92.6	
Diabetes									
Yes	1189	7.8	82	8.1	32	7.8	51	5.7	0.14
No	14085	92.2	929	91.9	380	92.2	846	94.3	
Aortic aneurysm									
Yes	147	1.0	11	1.1	0	0.0	4	0.5	0.08
No	15127	99.0	1000	98.9	412	100.0	893	99.6	
Immunosuppression									
Yes	162	1.1	19	1.9	4	1.0	10	1.1	0.12
No	15112	98.9	992	98.1	408	99.0	887	98.9	
Corticoids									
Yes	191	1.3	24	2.4	5	1.2	8	0.9	0.01
No	15083	98.8	987	97.6	407	98.8	889	99.1	
Smoking									
Yes	1963	12.9	136	13.5	67	16.3	110	12.3	0.19
No	13311	87.2	875	86.6	345	83.7	787	87.7	
Coagulopathy									
Yes	297	1.9	35	3.5	5	1.2	9	1.0	<.001
No	14977	98.1	976	96.5	407	98.8	888	99.0	
Antiplatelet medication									
Yes	1752	11.5	155	15.3	31	7.5	79	8.8	<.001
No	13522	88.5	856	84.7	381	92.5	818	91.2	
Anticoagulation therapy									
Yes	616	4.0	38	3.8	12	2.9	25	2.8	0.19
No	14658	95.9	973	96.2	400	97.1	872	97.2	

Unadjusted analysis identified a significantly higher value for recurrent repair with regard to almost all the target parameters, i.e., intraoperative complications, postoperative complications, complication-related reoperations, recurrences, pain at rest, pain on exertion, and pain requiring treatment on 1-year follow-up (Table 6). However, major differences were found in the recurrent repair results in relation to the previous operation. For example, in those cases where an open procedure was used for recurrent repair following endoscopic mesh repair in the primary operation, the intra- und postoperative complications, complication-related reoperation, and re-recurrence rates were comparable with those identified for open primary inguinal hernia repair.

**Table 6:** Perioperative and 1-year follow-up outcome in patients with open primary and recurrent unilateral inguinal hernia repair in men

	Primary		Previous suture		Previous open mesh		Previous endoscopic mesh		*p*
	*n*	%	*n*	%	*n*	%	*n*	%	
Intraoperative complications									0.03
Yes	158	1.0	13	1.3	10	2.4	14	1.6	
No	15116	99.0	998	98.7	402	97.6	883	98.4	
Postoperative complications									0.01
Yes	614	4.0	60	5.9	22	5.3	33	3.7	
No	14660	96.0	951	94.1	390	94.7	864	96.3	
Reoperation									0.02
Yes	181	1.2	21	2.1	9	2.2	9	1.0	
No	15093	98.8	990	97.9	403	97.8	888	99.0	
Recurrence									<0.001
Yes	113	0.7	17	1.7	20	4.6	10	1.1	
No	15161	99.3	994	98.3	392	95.2	887	98.9	
Pain at rest									<0.001
Yes	621	4.1	45	4.5	32	7.8	78	8.7	
No	14653	95.9	966	95.6	380	92.2	819	91.3	
Pain on exertion									<0.001
Yes	1291	8.5	92	9.1	53	12.9	135	15.0	
No	13983	91.6	919	90.9	359	87.1	762	85.0	
Chronic pain requiring treatment									<0.001
Yes	327	2.1	29	2.9	21	5.1	40	4.5	
No	14947	97.9	982	97.1	391	94.9	857	95.5	

### Multivariable analysis

#### Intraoperative complications

The multivariable analysis results for the intraoperative complication rate are illustrated in Table 7 (model matching: *p* < 0.001). The probability of onset of intraoperative complications during the primary and recurrent operations was significantly influenced by the risk factors (*p* = 0.01) and ASA score (*p* = 0.02). The presence of risk factors resulted in a significant increase in the intraoperative complication rate [OR = 1.534 (1.126–2.090)]. However, on comparing the implications of the various ASA scores, i.e., score II versus I and III/IV versus I, the higher scores were found to have exerted less influence on onset of intraoperative complications (Table 7). Open recurrent operations had a significant influence (*p* = 0.010), regardless of the previous operation, on the intraoperative complications compared with open primary operations. All recurrent operations were found to have negatively impacted onset of an intraoperative complication. The most negative influence was seen with an OR = 2.929 [1.515–5.664] with previous open mesh repair. The negative impact on the intraoperative complication rate was markedly less and comparable for previous endoscopic mesh repair and previous suture repair. In the open primary operations, too, the surgical technique had a significant influence (*p* < 0.001) on the occurrence of intraoperative complications. Compared with the Lichtenstein operation, the impact of the Plug and Gilbert techniques on onset of an intraoperative complication was markedly less, while that of the TIPP procedure was somewhat less.

**Table 7:** Intraoperative complications (model matching: *p* < 0.001)

Parameter	*p* value	Category	OR	95 % CI	
Open primary operation	<0.001	Gilbert versus Lichtenstein	0.134	0.033	0.549
		Plug versus Lichtenstein	0.175	0.077	0.398
		TIPP versus Lichtenstein	0.627	0.317	1.242
Risk factors	0.01	Yes versus no	1.534	1.126	2.090
Previous operation	0.01	Suture versus primary	1.330	0.750	2.359
		Endoscopic mesh versus primary	1.369	0.783	2.393
		Open mesh versus primary	2.929	1.515	5.664
ASA score	0.02	II versus I	0.637	0.446	0.912
		III/IV versus I	0.509	0.311	0.835
EHS classification lateral	0.16	Yes versus no	0.750	0.504	1.116
Defect size	0.25	II (1.5–3 cm) versus I (<1.5 cm)	0.929	0.587	1.472
		III (>3 cm) versus I (<1.5 cm)	1.221	0.746	1.996
BMI (5-point OR)	0.50		1.071	0.876	1.311
EHS classification medial	0.52	Yes versus no	0.876	0.583	1.315
EHS classification scrotal	0.56	Yes versus no	1.189	0.667	2.117
Age (10-year OR)	0.85		0.989	0.887	1.103
EHS classification femoral	0.97	Yes versus no	0.000	0.000	I

### Postoperative complications

The multivariable analysis results for the postoperative complication rate are shown in Table 8 (model matching: *p* < 0.001). The probability of postoperative complications was determined primarily by patient age (*p* < 0.001). Likewise, scrotal EHS classification (*p* < 0.001) and ASA score (*p* = 0.001) had a highly significant impact on the occurrence of postoperative complications. Higher age [10 year OR = 1.178 (1.107–1.255)], higher ASA score [III/IV vs I: OR = 1.237 (0.985–1.599)] as well as scrotal EHS classification [OR = 1.611 (1.215–2.136)] were conducive to onset of postoperative complications. Risk factors, too, significantly increased the probability of postoperative complications [OR = 1.268 (1.076–1.495)]. Unlike previous endoscopic repair, previous open suture repair [OR = 1.404 (1.062–1.855)] and mesh repair [OR = 1.444 (0.925–2.253)] increased the risk of onset of postoperative complications (*p* = 0.05) following the recurrent operation compared with open primary inguinal hernia repair. Conversely, a medial EHS classification [OR = 0.794 (0.647–0.974); *p* = 0.03] reduced the onset of postoperative complications. As regards the open primary operations, significant differences were also identified between the various surgical techniques (*p* < 0.001). Compared with the Lichtenstein operation, the Gilbert, Plug, and TIPP techniques presented a lower risk of postoperative complications.

**Table 8:** Postoperative complications (model matching: *p* < 0.001)

Parameter	*p* value	Category	OR	95 % CI	
Age (10-year OR)	<0.001		1.178	1.107	1.255
Open primary operation	<0.001	Gilbert versus Lichtenstein	0.338	0.189	0.606
		Plug versus Lichtenstein	0.849	0.658	1.095
		TIPP versus Lichtenstein	0.648	0.422	0.998
EHS classification scrotal	<0.001	Yes versus no	1.611	1.215	2.136
ASA score	0.001	II versus I	0.874	0.708	1.079
		III/IV versus I	1.237	0.958	1.599
Risk factors	0.005	Yes versus no	1.268	1.076	1.495
EHS classification medial	0.03	Yes versus no	0.794	0.647	0.974
Previous operation	0.05	Suture versus primary	1.404	1.062	1.855
		Endoscopic mesh versus primary	0.970	0.676	1.393
		Open mesh versus primary	1.444	0.925	2.253
EHS classification lateral	0.0	Yes versus no	0.818	0.664	1.007
Defect size	0.11	II (1.5–3 cm) versus I (<1.5 cm)	0.777	0.613	0.987
		III (>3 cm) versus I (<1.5 cm)	0.839	0.648	1.086
EHS classification femoral	0.73	Yes versus no	0.886	0.448	1.752
BMI (5-point OR)	0.96		0.997	0.894	1.113

### Complication-related reoperation

The multivariable analysis results for the complication-related reoperation rate are given in Table 9 (model matching: *p* < 0.001). This was influenced primarily by the ASA score (*p* < 0.001) and age (*p* = 0.002). A higher ASA score [III/IV vs I: OR = 2.200 (1.355–3.573)] and higher age [10 year OR = 1.213 [1.076–1.367)] significantly increased the reoperation risk. Likewise, the reoperation risk rose in the presence of risk factors [OR = 1.362 (1.015–1.827); *p* = 0.04]. Conversely, larger defect sizes (II vs I: OR 0.535 [0.359–0.798]; III vs I: OR = 0.701 [0.457–1.073]; *p* = 0.006) and medial EHS classification [OR = 0.695 (0.483–0.999); *p* = 0.05] reduced the reoperation risk. On comparing open primary inguinal hernia repair operations with recurrent repair, there was only tentative evidence of the recurrent repair results being less favorable. But major differences were identified in respect of the previous operations. While for open recurrent repair following previous endoscopic operation, no difference was found in terms of the risk assessment compared with open repair of primary inguinal hernias; for recurrent repair following previous open suture and mesh repair, a markedly higher risk of complication-related reoperation was identified. As regards the open primary procedures, no difference was found on comparing the Lichtenstein technique and Plug, Gilbert, and TIPP procedures.

**Table 9:** Complication-related reoperation (model matching: *p* < 0.001)

Parameter	*p* value	Category	OR	95 % CI	
ASA score	<.001	II versus I	1.037	0.673	1.596
		III/IV versus I	2.200	1.355	3.573
Age (10-year OR)	0.002		1.213	1.076	1.367
Defect size	0.006	II (1.5–3 cm) versus I (<1.5 cm)	0.535	0.359	0.798
		III (>3 cm) versus I (<1.5 cm)	0.701	0.457	1.073
Risk factors	0.04	Yes versus no	1.362	1.015	1.827
EHS medial	0.05	Yes versus no	0.695	0.483	0.999
Previous operation	0.05	Suture versus primary	1.600	1.008	2.539
		Endoscopic mesh versus primary	0.953	0.482	1.882
		Open mesh versus primary	2.048	1.024	4.096
Open primary operation	0.12	Gilbert versus Lichtenstein	0.538	0.217	1.333
		Plug versus Lichtenstein	0.641	0.374	1.100
		TIPP versus Lichtenstein	0.505	0.202	1.263
EHS classification scrotal	0.19	Yes versus no	1.396	0.846	2.304
EHS classification lateral	0.78	Yes versus no	0.947	0.647	1.386
BMI (5-point OR)	0.79		0.974	0.802	1.183
EHS classification femoral	0.90	Yes versus no	0.930	0.288	3.005

### Recurrence

Table 10 shows the multivariable analysis results for factors impacting recurrence on 1-year follow-up (model matching: *p* < 0.001). This was influenced essentially by the operation type (*p* < 0.001). Conduct of a recurrent operation resulted in a significantly higher risk of re-recurrence in comparison with an open primary repair of an inguinal hernia. That was true for each type of previous operation, albeit considerable differences were seen here. For example, whereas the probability of re-recurrence following previous endoscopic repair was only slightly higher than the risk posed by an open primary operation, the probability of re-recurrence associated with open recurrent repair following open suture repair, with an OR estimate 2.168 (1.290–3.644), and after previous open mesh repair with OR 7.032 (4.240–11.662), was considerably higher. Likewise, BMI had a highly significant impact on the recurrence rate on follow-up (*p* < 0.001). Accordingly, patients with a 5-point higher BMI had a higher recurrence rate [5-point OR = 1.438 (1.186–1.745)]. Higher age also gave rise to a higher recurrence risk (10 year OR = 1.170 [1.027–1.333], *p* = 0.02). For the primary operations, too, significant differences were found in the recurrence risk in relation to the surgical technique used (*p* = 0.02). For example, the risk of recurrence was lower following the Gilbert, Plug, and TIPP surgical procedures compared with the Lichtenstein operation.

**Table 10:** Recurrence rate on 1-year follow-up (model matching: *p* < 0.001)

Parameter	*p* value	Category	OR	95 % CI	
Previous operation	<0.001	Suture versus primary	2.168	1.290	3.644
		Endoscopic mesh versus primary	1.231	0.637	2.380
		Open mesh versus primary	7.032	4.240	11.662
BMI (5-point OR)	<0.001		1.438	1.186	1.745
Open primary operation	0.02	Gilbert versus Lichtenstein	0.475	0.172	1.310
		Plug versus Lichtenstein	0.535	0.298	0.962
		TIPP versus Lichtenstein	0.251	0.077	0.813
Age (10-year OR)	0.02		1.170	1.027	1.333
EHS classification lateral	0.08	Yes versus no	0.685	0.450	1.041
EHS classification medial	0.11	Yes versus no	1.443	0.925	2.251
Defect size	0.25	II (1.5–3 cm) versus I (<1.5 cm)	0.849	0.528	1.367
		III (>3 cm) versus I (<1.5 cm)	0.662	0.390	1.123
Risk factors	0.41	Yes versus no	0.863	0.607	1.226
EHS classification scrotal	0.60	Yes versus no	1.204	0.599	2.417
EHS classification femoral	0.82	Yes versus no	1.149	0.351	3.766
ASA score	0.99	II versus I	0.979	0.643	1.490
		III/IV versus I	0.986	0.570	1.705

### Pain at rest

The multivariable analysis results for pain at rest on 1-year follow-up are summarized in Table 11 (model matching: *p* < 0.001). This was affected primarily by the operation type (*p* < 0.001). A patient with an open recurrent operation had a significantly higher risk of pain in comparison with a patient with open primary repair. But here, too, differences were found in the OR estimate of pain at rest rate following recurrent repair in relation to the previous operation. For example, the risk of pain following recurrent repair after previous open mesh [OR = 2.112 (1.449–3.077)] and endoscopic mesh repair [OR = 1.895 (1.473–2.438)] was markedly higher than after previous suture repair [OR = 1.095 (0.801–1.495)]. Likewise, a higher BMI presented a significant risk of onset of pain at rest [5-point OR = 1.236 (1.120–1.364); *p* < 0.001]. A larger hernia defect [II vs I: OR = 0.716 (0.580–0.883); III vs I: OR = 0.684 (0.540–0.867); *p* = 0.003], a higher age [10-year OR = 0.930 (0.880–0.982); *p* = 0.009], scrotal [OR = 0.599 (0.399–0.898); *p* = 0.01], and femoral hernia [OR = 0.349 (0.182–0.946); *p* = 0.04] reduced the risk of onset of pain at rest.

**Table 11:** Pain at rest on 1-year follow-up (model matching: *p* < 0.001)

Parameter	*p* value	Category	OR	95 % CI	
Previous operation	<0.001	Suture versus primary	1.095	0.801	1.495
		Endoscopic mesh versus primary	1.895	1.473	2.438
		Open mesh versus primary	2.112	1.449	3.077
BMI (5-point OR)	<0.001		1.236	1.120	1.364
Open primary operation	<0.001	Gilbert versus Lichtenstein	0.722	0.497	1.048
		Plug versus Lichtenstein	0.654	0.511	0.836
		TIPP versus Lichtenstein	0.470	0.307	0.718
Defect size	0.003	II (1.5–3 cm) versus I (<1.5 cm)	0.716	0.580	0.883
		III (>3 cm) versus I (<1.5 cm)	0.684	0.540	0.867
Age (10-year OR)	0.009		0.930	0.880	0.982
EHS classification scrotal	0.01	Yes versus no	0.599	0.399	0.898
EHS classification femoral	0.04	Yes versus no	0.349	0.128	0.946
EHS classification lateral	0.35	Yes versus no	0.906	0.739	1.112
Risk factors	0.41	Yes versus no	1.070	0.910	1.259
EHS classification medial	0.48	Yes versus no	1.076	0.877	1.320
ASA score	0.66	II versus I	0.960	0.799	1.153
		III/IV versus I	0.888	0.685	1.151

In the primary operations, too, significant differences (*p* < 0.001) favorable to the Gilbert, Plug, und TIPP procedures were seen compared with the Lichtenstein operation.

### Pain on exertion

Table 12 shows the multivariable analysis results for pain on exertion on follow-up (model matching: *p* < 0.001). As for pain at rest, pain on exertion also occurred more often after recurrent operations and in patients with higher BMI (in each case *p* < 0.001). For the recurrent operations, the risk of that happening also depended on the previous operation. Compared with primary operation, the risk of onset of pain on exertion following recurrent repair was higher when endoscopic repair [OR = 1.590 (1.306–1.936)] or open mesh repair [OR = 1.577 (1.168–2.129)] was used in the previous operation compared with suture repair in the previous operation [OR = 1.077 (0.860–1.349)]. A lower risk of pain on exertion was identified for patients with a higher age [OR = 0.856 (0.824–0.890); *p* < 0.001], scrotal hernia [OR = 0.507 (0.371–0.692); *p* < 0.001], lateral hernia [OR = 0.809 (0.695–0.942); *p* = 0.006], and femoral hernia [OR = 0.452 (0.244–0.837); *p* = 0.01]. As regards the open primary inguinal hernia operations, significant differences unfavorable to the Lichtenstein technique (*p* < 0.001) were observed once again.

**Table 12:** Pain on exertion on 1-year follow-up (model matching: *p* < 0.001)

Parameter	*p* value	Category	OR	95 % CI	
Age (10-year OR)	<0.001		0.856	0.824	0.890
Defect size	<0.001	II (1.5–3 cm) versus I (<1.5 cm)	0.752	0.647	0.874
		III (>3 cm) versus I (<1.5 cm)	0.565	0.475	0.673
Previous operation	<0.001	Suture versus primary	1.077	0.860	1.349
		Endoscopic mesh versus primary	1.590	1.306	1.936
		Open mesh versus primary	1.577	1.168	2.129
BMI (5-point OR)	<0.001		1.183	1.099	1.274
EHS classification scrotal	<0.001	Yes versus no	0.507	0.371	0.692
Open primary operation	<0.001	Gilbert versus Lichtenstein	0.656	0.495	0.871
		Plug versus Lichtenstein	0.748	0.633	0.883
		TIPP versus Lichtenstein	0.731	0.560	0.954
EHS classification lateral	0.006	Yes versus no	0.809	0.695	0.942
EHS classification femoral	0.01	Yes versus no	0.452	0.244	0.837
ASA score	0.03	II versus I	1.044	0.916	1.190
		III/IV versus I	0.844	0.695	1.025
Risk factors	0.13	Yes versus no	1.096	0.975	1.233
EHS classification medial	0.52	Yes versus no	0.951	0.818	1.107

### Chronic pain requiring treatment

The multivariable analysis results for chronic pain requiring treatment are presented in Table 13 (model matching: *p* < 0.001). Again, this was highly influenced by the operation type (*p* < 0.001). An open recurrent operation in comparison to open primary repair increased onset of chronic pain requiring treatment. But here, too, that risk was dependent on the previous operation. As for pain at rest and on exertion, the impact of the previous operation on onset of chronic pain requiring treatment was more unfavorable following endoscopic [OR = 1.759 (1.249–2.477)] and open [OR = 2.274 (1.434–3.608)] mesh procedures than after open suture repair [OR = 1.290 (0.875–1.903)]. Likewise, a higher BMI, as in the case of pain at rest and on exertion, negatively impacted chronic pain requiring treatment [OR = 1.276 (1.125–1.448); *p* < 0.001]. By contrast, a high age [10-year OR = 0.875 (0.813–0.942); *p* < 0.001] and larger defect size [II vs I: OR = 0.783 (0.588–1.044); III vs I: OR = 0.619 (0.447–0.858); *p* = 0.01] reduced the risk of chronic pain requiring treatment. For the open primary techniques, no significant differences were seen here between the various surgical procedures.

**Table 13:** Chronic pain requiring treatment (model matching: *p* < 0.001)

Parameter	*p* value	Category	OR	95 % CI	
Previous operation	<0.001	Suture versus primary	1.290	0.875	1.903
		Endoscopic mesh versus primary	1.759	1.249	2.477
		Open mesh versus primary	2.274	1.434	3.608
BMI (5-point OR)	<0.001		1.276	1.125	1.448
Age (10-year OR)	<0.001		0.875	0.813	0.942
Defect size	0.01	II (1.5–3 cm) versus I (<1.5 cm)	0.783	0.588	1.044
		III (>3 cm) versus I (<1.5 cm)	0.619	0.447	0.858
EHS classification scrotal	0.05	Yes versus no	0.568	0.320	1.009
ASA score	0.07	II versus I	1.330	1.031	1.716
		III/IV versus I	1.428	1.005	2.030
EHS classification lateral	0.08	Yes versus no	0.785	0.600	1.027
EHS classification medial	0.17	Yes versus no	1.217	0.921	1.608
Open primary operation	0.23	Gilbert versus Lichtenstein	0.700	0.410	1.196
		Plug versus Lichtenstein	0.749	0.542	1.037
		TIPP versus Lichtenstein	0.885	0.561	1.399
Risk factors	0.24	Yes versus no	1.139	0.918	1.412
EHS classification femoral	0.96	Yes versus no	1.024	0.447	2.349

## Discussion

This analysis based on data from the Herniamed Registry compares 15,274 patients who had undergone an open primary inguinal hernia operation versus 2320 patients with open repair of a recurrent hernia following a previous open suture, open mesh, or endoscopic mesh operation. All patients had been followed up for 1 year. The target variables applied were the intra- and postoperative complication rates, complication-related reoperation rates, recurrence or re-recurrence rates, the rates of pain at rest and on exertion, as well as chronic pain requiring treatment. To gain a clearer picture of how the different influence variables impacted the results, the open recurrent operation group was compared with the open primary operation group in relation to the procedure used in the previous operation. In the open primary operation group, the influence exerted by the other open techniques (Gilbert, Plug, and TIPP) used as an alternative to the Lichtenstein operation was analyzed.

As regards the intraoperative complication rate, multivariable analysis demonstrated that the recurrent operation was an independent influence factor for onset of intraoperative complications. That impact was least favorable following not only previous open mesh operation, but was also observed after open suture repair and endoscopic mesh repair.

Conversely with regard to the postoperative complications, open recurrent repair following previous endoscopic repair was not found to be associated with any higher risk compared with open primary inguinal hernia operation. However, significantly more postoperative complications must be anticipated after recurrent operation following previous open suture or mesh repair.

This trend is also reflected in the complication-related reoperation rates.

The impact of recurrent inguinal hernia repair compared with primary operation is particularly pronounced with regard to the recurrence and re-recurrence rates. Here, too, the previous operation plays a dominant role. While open recurrent repair following previous endoscopic operation compared with open primary inguinal hernia repair presents only a slightly higher risk for onset of re-recurrence, the risk following previous open suture repair is markedly higher, and after previous open mesh repair, it is extremely higher.

Likewise for pain at rest, pain on exertion and chronic pain requiring treatment, open recurrent repair compared with the primary operation is an independent significant risk factor for an unfavorable outcome. Surprisingly, here the least negative effect was observed for open recurrent repair following previous open suture repair. Both previous open mesh repair and previous endoscopic repair constitute highly significant influence factors for onset of pain following open recurrent repair.

Here one would have expected that the risk would be lower after previous endoscopic operation since repair is carried out in a new plane of dissection. However, here previous open suture repair presented the least risk for occurrence of pain after open recurrent repair.

For the findings presented here, one must also take into account that analysis of open primary inguinal hernia operations also revealed significant differences in the results obtained for the various surgical techniques. For example, multivariable analysis identified significantly better results for the open Gilbert, Plug, and TIPP techniques compared with the Lichtenstein operation.

In summary, analysis of the data from the Herniamed Hernia Registry as presented here demonstrates that, taking into account all other influence factors, open recurrent repair compared with open primary inguinal hernia repair is associated with a significantly poorer perioperative and 1-year follow-up outcome. However, relevant differences were identified in relation to the technique employed for the primary operation, and to the type of operation used prior to recurrent repair. The data presented here confirm the recommendation given in the Guidelines of the European Hernia Society to use open repair in a new plane of dissection for recurrent repair following previous endoscopic repair of a primary inguinal hernia. The results demonstrate that the risk for onset of a postoperative complication, complication-related reoperation, and re-recurrence is comparable with that presented by the primary operation. Open recurrent repair after previous open mesh repair posed the highest risk for occurrence of intraoperative and postoperative complications, complicated-related reoperations, re-recurrences, pain at rest, pain on exertion, and chronic pain requiring treatment. Therefore, the recommendation in the Guidelines of the European Hernia Society should definitely be observed for recurrent repair following previous open mesh repair.

## Appendix

### Herniamed Study Group

#### Scientific Board

Köckerling, Ferdinand (Chairman) (Berlin); Berger, Dieter (Baden–Baden); Bittner, Reinhard (Rottenburg); Bruns, Christiane (Magdeburg); Dalicho, Stephan (Magdeburg); Fortelny, René (Wien); Jacob, Dietmar (Berlin); Koch, Andreas (Cottbus); Kraft, Barbara (Stuttgart); Kuthe, Andreas (Hannover); Lippert, Hans (Magdeburg): Lorenz, Ralph (Berlin); Mayer, Franz (Salzburg); Moesta, Kurt Thomas (Hannover); Niebuhr, Henning (Hamburg); Peiper, Christian (Hamm); Pross, Matthias (Berlin); Reinpold, Wolfgang (Hamburg); Simon, Thomas (Sinsheim); Stechemesser, Bernd (Köln); Unger, Solveig (Chemnitz)

#### Participants

Ahmetov, Azat (Saint-Petersburg); Alapatt, Terence Francis (Frankfurt/Main); Anders, Stefan (Berlin); Anderson, Jürina (Würzburg); Arndt, Anatoli (Elmshorn); Asperger, Walter (Halle); Avram, Iulian (Saarbrücken); Barkus; Jörg (Velbert); Becker, Matthias (Freital); Behrend, Matthias (Deggendorf); Beuleke, Andrea (Burgwedel); Berger, Dieter (Baden–Baden); Bittner, Reinhard (Rottenburg); Blaha, Pavel (Zwiesel); Blumberg, Claus (Lübeck); Böckmann, Ulrich (Papenburg); Böhle, Arnd Steffen (Bremen); Böttger, Thomas Carsten (Fürth); Bolle, Ludger (Berlin); Borchert, Erika (Grevenbroich); Born, Henry (Leipzig); Brabender, Jan (Köln); Brauckmann, Markus (Rüdesheim am Rhein); Breitenbuch von, Philipp (Radebeul); Brüggemann, Armin (Kassel); Brütting, Alfred (Erlangen); Budzier, Eckhard (Meldorf); Burghardt, Jens (Rüdersdorf); Carus, Thomas (Bremen); Cejnar, Stephan-Alexander (München); Chirikov, Ruslan (Dorsten); Comman, Andreas (Bogen); Crescenti, Fabio (Verden/Aller); Dapunt, Emanuela (Bruneck); Decker, Georg (Berlin); Demmel, Michael (Arnsberg); Descloux, Alexandre (Baden); Deusch, Klaus-Peter (Wiesbaden); Dick, Marcus (Neumünster); Dieterich, Klaus (Ditzingen); Dietz, Harald (Landshut); Dittmann, Michael (Northeim); Dornbusch, Jan (Herzberg/Elster); Drummer, Bernhard (Forchheim); Eckermann, Oliver (Luckenwalde); Eckhoff, Jörn/Hamburg); Elger, Karlheinz (Germersheim); Engelhardt, Thomas (Erfurt); Erichsen, Axel (Friedrichshafen); Eucker, Dietmar (Bruderholz); Fackeldey, Volker (Kitzingen); Farke, Stefan (Delmenhorst); Faust, Hendrik (Emden); Federmann, Georg (Seehausen); Feichter, Albert (Wien); Fiedler, Michael (Eisenberg); Fischer, Ines (Wiener Neustadt); Fortelny, René H. (Wien); Franczak, Andreas (Wien); Franke, Claus (Düsseldorf); Frankenberg von, Moritz (Salem); Frehner, Wolfgang (Ottobeuren); Friedhoff, Klaus (Andernach); Friedrich, Jürgen (Essen); Frings, Wolfram (Bonn); Fritsche, Ralf (Darmstadt); Frommhold, Klaus (Coesfeld); Frunder, Albrecht (Tübingen); Fuhrer, Günther (Reutlingen); Gassler, Harald (Villach); Gawad, Karim A. Frankfurt/Main); Gerdes, Martin (Ostercappeln); Germanov, German (Halberstadt; Gilg, Kai-Uwe (Hartmannsdorf); Glaubitz, Martin (Neumünster); Glutig, Holger (Meissen); Gmeiner, Dietmar (Bad Dürrnberg); Göring, Herbert (München); Grebe, Werner (Rheda-Wiedenbrück); Grothe, Dirk (Melle); Gürtler, Thomas (Zürich); Hache, Helmer (Löbau); Hämmerle, Alexander (Bad Pyrmont); Haffner, Eugen (Hamm); Hain, Hans-Jürgen (Gross-Umstadt); Hammans, Sebastian (Lingen); Hampe, Carsten (Garbsen); Harrer, Petra (Starnberg); Heinzmann, Bernd (Magdeburg); Heise, Joachim Wilfried (Stolberg); Heitland, Tim (München); Helbling, Christian (Rapperswil); Hempen, Hans-Günther (Cloppenburg); Henneking, Klaus-Wilhelm (Bayreuth); Hennes, Norbert (Duisburg); Hermes, Wolfgang (Weyhe); Herrgesell, Holger (Berlin); Herzing, Holger Höchstadt); Hessler, Christian (Bingen); Hildebrand, Christiaan (Langenfeld); Höferlin, Andreas (Mainz); Hoffmann, Henry (Basel); Hoffmann, Michael (Kassel); Hofmann, Eva M. (Frankfurt/Main); Hopfer, Frank (Eggenfelden); Hornung, Frederic (Wolfratshausen); Hügel, Omar (Hannover); Hüttemann, Martin (Oberhausen); Huhn, Ulla (Berlin); Hunkeler, Rolf (Zürich); Imdahl, Andreas (Heidenheim); Jacob, Dietmar (Berlin); Jenert, Burghard (Lichtenstein); Jugenheimer, Michael (Herrenberg); Junger, Marc (München); Kaaden, Stephan (Neustadt am Rübenberge); Käs, Stephan (Weiden); Kahraman, Orhan (Hamburg); Kaiser, Christian (Westerstede); Kaiser, Stefan (Kleinmachnow); Kapischke, Matthias (Hamburg); Karch, Matthias (Eichstätt); Kasparek, Michael S. (München); Keck, Heinrich (Wolfenbüttel); Keller, Hans W. (Bonn); Kienzle, Ulrich (Karlsruhe); Kipfmüller, Brigitte (Köthen); Kirsch, Ulrike (Oranienburg); Klammer, Frank (Ahlen); Klatt, Richard (Hagen); Kleemann, Nils (Perleberg); Klein, Karl-Hermann (Burbach); Kleist, Sven (Berlin); Klobusicky, Pavol (Bad Kissingen); Kneifel, Thomas (Datteln); Knoop, Michael (Frankfurt/Oder); Knotter, Bianca (Mannheim); Koch, Andreas (Cottbus); Köckerling, Ferdinand (Berlin); Köhler, Gernot (Linz); König, Oliver (Buchholz); Kornblum, Hans (Tübingen); Krämer, Dirk (Bad Zwischenahn); Kraft, Barbara (Stuttgart); Kreissl, Peter (Ebersberg); Krones, Carsten Johannes (Aachen); Kruse, Christinan (Aschaffenburg); Kube, Rainer (Cottbus); Kühlberg, Thomas (Berlin); Kuhn, Roger (Gifhorn); Kusch, Eduard (Gütersloh); Kuthe, Andreas (Hannover); Ladberg, Ralf (Bremen); Ladra, Jürgen (Düren); Lahr-Eigen, Rolf (Potsdam); Lainka, Martin (Wattenscheid); Lammers, Bernhard J. (Neuss); Lancee, Steffen (Alsfeld); Lange, Claas (Berlin); Laps, Rainer (Ehringshausen); Larusson, Hannes Jon (Pinneberg); Lauschke, Holger (Duisburg); Leher, Markus (Schärding); Leidl, Stefan (Waidhofen/Ybbs); Lenz, Stefan (Berlin); Lesch, Alexander (Kamp-Lintfort); Liedke, Marc Olaf (Heide); Lienert, Mark (Duisburg); Limberger, Andreas (Schrobenhausen); Limmer, Stefan (Würzburg); Locher, Martin (Kiel); Loghmanieh, Siawasch (Viersen); Lorenz, Ralph (Berlin); Mallmann, Bernhard (Krefeld); Manger, Regina (Schwabmünchen); Maurer, Stephan (Münster); Mayer, Franz (Salzburg); Mellert, Joachim (Höxter); Menzel, Ingo (Weimar); Meurer, Kirsten (Bochum); Meyer, Moritz (Ahaus); Mirow, Lutz (Kirchberg); Mittenzwey, Hans-Joachim (Berlin); Mörder-Köttgen, Anja (Freiburg); Moesta, Kurt Thomas (Hannover); Moldenhauer, Ingolf (Braunschweig); Morkramer, Rolf (Xanten); Mosa, Tawfik (Merseburg); Müller, Hannes (Schlanders); Münzberg, Gregor (Berlin); Mussack, Thomas (St. Gallen); Neumann, Jürgen (Haan); Neumeuer, Kai (Paderborn); Niebuhr, Henning (Hamburg); Nix, Carsten (Walsrode); Nölling, Anke (Burbach); Nostitz, Friedrich Zoltán (Mühlhausen); Obermaier, Straubing); Öz-Schmidt, Meryem (Hanau); Oldorf, Peter (Usingen); Olivieri, Manuel (Pforzheim); Pawelzik, Marek (Hamburg); Peiper, Christian (Hamm); Peitgen, Klaus (Bottrop); Pertl, Alexander (Spittal/Drau); Philipp, Mark (Rostock); Pickart, Lutz (Bad Langensalza); Pizzera, Christian (Graz); Pöllath, Martin (Sulzbach-Rosenberg); Possin, Ulrich (Laatzen); Prenzel, Klaus (Bad Neuenahr-Ahrweiler); Pröve, Florian (Goslar); Pronnet, Thomas (Fürstenfeldbruck); Pross, Matthias (Berlin); Puff, Johannes (Dinkelsbühl); Rabl, Anton (Passau); Rapp, Martin (Neunkirchen); Reck, Thomas (Püttlingen); Reinpold, Wolfgang (Hamburg); Reuter, Christoph (Quakenbrück); Richter, Jörg (Winnenden); Riemann, Kerstin (Alzenau-Wasserlos); Rodehorst, Anette (Otterndorf); Roehr, Thomas (Rödental); Roncossek, Bremerhaven); Roth Hartmut (Nürnberg); Sardoschau, Nihad (Saarbrücken); Sauer, Gottfried (Rüsselsheim); Sauer, Jörg (Arnsberg); Seekamp, Axel (Freiburg); Seelig, Matthias (Bad Soden); Seidel, Hanka (Eschweiler); Seiler, Christoph Michael (Warendorf); Seltmann, Cornelia (Hachenburg); Senkal, Metin (Witten); Shamiyeh, Andreas (Linz); Shang, Edward (München); Siemssen, Björn (Berlin); Sievers, Dörte (Hamburg); Silbernik, Daniel (Bonn); Simon, Thomas (Sinsheim); Sinn, Daniel (Olpe); Sinning, Frank (Nürnberg); Smaxwil, Constatin Aurel (Stuttgart); Schabel, Volker (Kirchheim/Teck); Schadd, Peter (Euskirchen); Schassen von, Christian (Hamburg); Schattenhofer, Thomas (Vilshofen); Scheidbach, Hubert (Neustadt/Saale); Schelp, Lothar (Wuppertal); Scherf, Alexander (Pforzheim); Scheyer, Mathias (Bludenz); Schimmelpenning, Hendrik (Neustadt in Holstein); Schinkel, Svenja (Kempten); Schmid, Michael (Gera); Schmid, Thomas (Innsbruck); Schmidt, Rainer (Paderborn); Schmidt, Sven-Christian (Berlin); Schmidt, Ulf (Mechernich); Schmitz, Heiner (Jena); Schmitz, Ronald (Altenburg); Schöche, Jan (Borna); Schoenen, Detlef (Schwandorf); Schrittwieser, Rudolf/Bruck an der Mur); Schroll, Andreas (München); Schultz, Christian (Bremen-Lesum); Schultz, Harald (Landstuhl); Schulze, Frank P. Mülheim an der Ruhr); Schumacher, Franz-Josef (Oberhausen); Schwab, Robert (Koblenz); Schwandner, Thilo (Lich); Schwarz, Jochen Günter (Rottenburg); Schymatzek, Ulrich (Radevormwald); Spangenberger, Wolfgang (Bergisch-Gladbach); Sperling, Peter (Montabaur); Staade, Katja (Düsseldorf); Staib, Ludger (Esslingen); Stamm, Ingrid (Heppenheim); Stark, Wolfgang (Roth); Stechemesser, Bernd (Köln); Steinhilper, Uz (München); Stengl, Wolfgang (Nürnberg); Stern, Oliver (Hamburg); Stöltzing, Oliver (Meißen); Stolte, Thomas (Mannheim); Stopinski, Jürgen (Schwalmstadt); Stubbe, Hendrik (Güstrow/); Stülzebach, Carsten (Friedrichroda); Tepel, Jürgen (Osnabrück); Terzić, Alexander (Wildeshausen); Teske, Ulrich (Essen); Thews, Andreas (Schönebeck); Tichomirow, Alexej (Brühl); Tillenburg, Wolfgang (Marktheidenfeld); Timmermann, Wolfgang (Hagen); Tomov, Tsvetomir (Koblenz; Train, Stefan H. (Gronau); Trauzettel, Uwe (Plettenberg); Triechelt, Uwe (Langenhagen); Ulcar, Heimo (Schwarzach im Pongau); Unger, Solveig (Chemnitz); Verweel, Rainer (Hürth); Vogel, Ulrike (Berlin); Voigt, Rigo (Altenburg); Voit, Gerhard (Fürth); Volkers, Hans-Uwe (Norden); Vossough, Alexander (Neuss); Wallasch, Andreas (Menden); Wallner, Axel (Lüdinghausen); Warscher, Manfred (Lienz); Warwas, Markus (Bonn); Weber, Jörg (Köln); Weihrauch, Thomas (Ilmenau); Weiß, Johannes (Schwetzingen); Weißenbach, Peter (Neunkirchen); Werner, Uwe (Lübbecke-Rahden); Wessel, Ina (Duisburg); Weyhe, Dirk (Oldenburg); Wieber, Isabell (Köln); Wiesmann, Aloys (Rheine); Wiesner, Ingo (Halle); Withöft, Detlef (Neutraubling); Woehe, Fritz (Sanderhausen); Wolf, Claudio (Neuwied); Yaksan, Arif (Wermeskirchen); Yildirim, Selcuk (Berlin); Zarras, Konstantinos (Düsseldorf); Zeller, Johannes (Waldshut-Tiengen); Zhorzel, Sven (Agatharied); Zuz, Gerhard (Leipzig);
